# NK Cells and Type 1 Diabetes

**DOI:** 10.1080/17402520600877182

**Published:** 2006

**Authors:** Melanie Rodacki, Adolpho Milech, José Egídio Paulo de Oliveira

**Affiliations:** 1 Diabetes and Nutrology section Federal University of Rio de Janeiro (UFRJ) Rio de Janeiro RJ Brazil; 2 State Institute of Diabetes and Endocrinology (IEDE) Rio de Janeiro RJ Brazil

## Abstract

Type 1 diabetes (T1D) is characterized by an immuno-mediated progressive destruction of the pancreatic β cells. Due to the ability of NK cells to kill target cells as well as to interact with antigen-presenting and T cells, it has been suggested that they could be involved in one or multiple steps of the immune-mediated attack that leads to T1D. Abnormalities in the frequency and activity of NK cells have been described both in animal models and patients with T1D. Some of these alterations are linked to its onset while others seem to be a consequence of the disease. Here, we discuss the main characteristics of NK cells and review the studies that investigated the role of NK cells in T1D, both in mouse models and humans.


*Clinical & Developmental Immunology*, June–December 2006; 13(2–4): 101–107

Taylor & Francis

Taylor & Francis Group

### NK cells and type 1 diabetes

MELANIE RODACKI[1,2], ADOLPHO MILECH[1], & JOSÉ EGÍDIO PAULO DE OLIVEIRA[1]

[1]*Diabetes and Nutrology section, Federal University of Rio de Janeiro (UFRJ), Rio de Janeiro, RJ, Brazil, and* [2]*State Institute of Diabetes and Endocrinology (IEDE), Rio de Janeiro, RJ, Brazil*

**Abstract**
Type 1 diabetes (T1D) is characterized by an immuno-mediated progressive destruction of the pancreatic β cells. Due to the ability of NK cells to kill target cells as well as to interact with antigen-presenting and T cells, it has been suggested that they could be involved in one or multiple steps of the immune-mediated attack that leads to T1D. Abnormalities in the frequency and activity of NK cells have been described both in animal models and patients with T1D. Some of these alterations are linked to its onset while others seem to be a consequence of the disease. Here, we discuss the main characteristics of NK cells and review the studies that investigated the role of NK cells in T1D, both in mouse models and humans.

**Keywords:** *Type 1 diabetes, T cells, NK cells*

### Introduction

Type 1 diabetes (T1D) is characterized by an immuno-mediated progressive destruction of the pancreatic β cells (Kelly et al. 2003). Although the precise mechanisms involved in its pathogenesis are still unclear, it is known that autoreactive T cells have a central role in the process. Due to the ability of NK cells to kill target cells as well as to interact with antigen-presenting and T cells, it has been suggested that they could be involved in one or multiple steps of the immune-mediated attack that leads to T1D. Here, we discuss the main characteristics of NK cells and review the studies that investigated the role of NK cells in T1D, both in mouse models and humans.

### NK cells

NK cells are large granular lymphocytes that do not express B or T cell receptors and participate in the innate immune response. Besides playing important roles in defence against malignancies and infectious diseases, NK cells are implicated in the graft-versus-host disease, the regulation of hematopoiesis and are capable of interfering in the adaptative immune response (Flodstrom et al. 2002). Under normal circumstances, they are primarily located in the peripheral blood, bone marrow, spleen and liver (Moretta et al. 2002; French and Yokoyama 2004; Sinkovics and Horvath 2005). In humans, NK cells are identified by the lack of the surface marker CD3 and the presence of CD56 with or without CD16.

NK cells act through the cytotoxic destruction of their target cells. Unlike cytolitic T lymphocytes, they exert their effector function without the need for previous *in vitro* or *in vivo* activation. This characteristic makes them remarkably suited to mediate the first line of defence against pathogens, as part of the innate immune response (Baxter and Smyth 2002; Colucci et al. 2003). When activated, NK cells induce apoptosis of the target cells mainly through the exocytosis of perforin and granzyme. They also secrete pro-inflammatory cytokines such as interferon gamma (IFNg), tumor necrosis factor (TNF), granulocyte–macrophage colony-stimulating factor (GM-CSF) and macrophage inflammatory protein 1α and 1β (Backstrom et al. 2004).

Correspondence: M. Rodacki, Diabetes and Nutrology Section, Federal University of Rio de Janeiro (UFRJ), Rua Maria Angélica 46 apto 402, Lagoa, Rio de Janerio, RJ 22470-200, Brazil. E-mail: mrodacki@hucff.ufrj.br

ISSN 1740-2522 print/ISSN 1740-2530 online © 2006 Taylor & Francis
DOI: 10.1080/17402520600877182

NK cells recognize the target cells through specific molecules on the surface of these cells. These molecules may be either inadequately present or absent due to cell infection or its transformation. In the first situation, this could be due to non-self molecules encoded by pathogens or, alternatively, self-molecules only expressed in a higher frequency in certain adverse conditions. The second scenario implies the absence of molecules normally present on the surface of the autologous cells, like MHC class I. These alterations could render these cells susceptible to NK lysis (Raulet 2004). Generally, this second mechanism alone does not trigger cytotoxicity, unless it is combined to the anomalous expression of other molecules on the target cell surface.

There are receptors on the surface of the NK cells that can trigger cell stimulation or inhibition. Briefly, these receptors are coupled with intra-cellular signalling adapters that contain activation or inhibition sites based on their tyrosine residues. These are called, respectively, immunoreceptor tyrosine-based activation motifs (ITAM) e immune tyrosine based inhibitory motifs (ITIM) (Lanier and Bakker 2000).

Two main categories of NK inhibitory receptors were identified in humans: the heterodimer CD94:NKG2A, specific for HLA E molecules; and the killer cell immunoglobulin-like (KIR) receptors, that recognize HLA A, B and C molecules. KIR receptors, however, are not always inhibitory. They belong to a diverse family of receptors, both stimulatory or inhibitory, codified by genes in the chromosome 19 and expressed mainly by NK cells, but also by some subgroups of T lymphocytes (Vilches and Parham 2002; Middleton et al. 2005). Based on the combination of KIR genes, two major groups of haplotypes can be defined, A and B. While genes encoding inhibitory receptors predominate in the former, two or more activating KIR genes are present in the latter (Slik et al. 2003).

NKG2D and natural cytotoxicity receptors (NCRs) are the main NK activating receptors. NCRs (NKp30, NKp44, NKp46 and NKp80) are expressed exclusively in NK cells and belong to the superfamily of immunoglobulins (Pende et al. 1999; Sivori et al. 1999; Moretta and Moretta 2004). Their ligands are not completely defined, but it is known that at least part of them (NKp44 and NKp46) recognize viral hemmaglutinins. Heparan sulphate proteoglycans have also been shown to act as ligands for NKp46 and NKp30 (Arnon et al. 2001; Mandelboim et al. 2001; Bloushtain et al. 2004). The NKG2D receptor is expressed by the majority of human NK cells, but also in other lymphocytes such as CD8+. It is codified by the NK receptor gene complex in chromosome 12. Differently from the other NKG2 receptors, which are inhibitory and form dimers with the receptor CD94, the receptor NKG2D is a homodimer that recognizes a variety of ligands induced by stress, such as non-classic HLA class I molecules MICA and MICB, as well as ULBPs (Raulet 2003; Andre et al. 2004).

Although numerous receptors have been implicated in NK cells activation, it is not known if any specific receptor alone is capable of triggering the NK effector function by itself. A synergic interaction between multiple receptors seems to be necessary for triggering the cytotoxicity and cytokines production. Therefore, NK cell function depends on the balance of various signals from stimulatory and inhibitory receptors, as well as the expression of their corresponding ligands (Hoffman 1980).

### NK cells in autoimmune diseases

Aproximately 5% of the population in the Western countries are affected by autoimmune diseases (Flodstrom et al. 2002). Although there is a strong genetic component determining susceptibility to these diseases, some kind of environmental factor is usually also necessary to trigger their appearance. Under normal conditions, self-tolerance mechanisms prevent intra-thymic maturation and activation of autoreactive lymphocytes, what is called central tolerance (Janeway et al. 2005). However, a small proportion of the autoreactive cells escapes from this process, undergoes maturation and reaches the peripheral circulation. To prevent the autologous cells against destruction by these autoreactive cells, there are also peripheral mechanisms that can prevent the action of these autoreactive or even destroy them, what is known as peripheral tolerance. When there is a failure in the central or peripheral self-tolerance mechanisms, immune reaction to self-antigens can occur, leading to autoimmune diseases.

NK cells might have a direct influence in the development of autoimmune diseases, destroying cells in their target organs, or, alternatively, have an indirect role, regulating the adaptative immune response (Kos and Engeman 1996; Shi et al. 2000). This modulation could occur in various steps of the autoimmune response. Due to their potential to interact with antigen-presenting cells (like dendritic cells), NK cells could interfere in the priming of autoimmune responses. They might also influence the downstream response, as it is known that they can affect the proliferation and generation of B and T autoreactive lymphocytes. Although NK cells might prevent the presentation of self-antigens by dendritic cells, avoiding the autoimmune response, they also seem to be necessary for the initiation of auto-reactive B and/or T cell response (Sinkovics and Horvath 2005). On the other hand, NK cells can also secrete cytokines that supress B and T cell responses or even destroy them (French and Yokoyama 2004).

Given their cytololytic ability, NK cells have been potentially involved in the pathogenesis of diseases caused by tissue destruction, as are most autoimmune diseases. It has been shown that NK cells can kill autologous cells (Hansson et al. 1981; Morse et al. 2001). However, under normal conditions, molecules on the surface of the autologous cells, especially MHC class I, engage inhibitory receptors on the surface of NK cells preventing them from delivering a lytic signal and making them self-tolerant. Loss of self-tolerance can occur if an autologous cell loses the expression of MHC class I (Flodstrom et al. 2002). The exact mechanism involved in the development of self-tolerance by the NK cells is not completely understood.

NK cells have been identified in target organs of patients suffering from autoimmune diseases (Flodstrom et al. 2002). Although this phenomenon might be explained solely by the migration of NK cells as part of any inflammatory process, independently of its cause, there is evidence that NK cells are capable of attacking autologous cells (Hansson et al. 1981; MacKay et al. 1986; Nakamura et al. 1990; Morse et al. 2001) which may indicate that they could contribute to the development of autoimmune cell destruction. As infectious disease have been implicated as potential triggers for many autoimmune diseases and one of the main roles of NK cells is the protection against infections, a link between NK cells and autoimmune diseases would seem reasonable. NK cells might either promote or suppress autoimmune diseases triggered by infectious diseases. By rapidly clearing the infection, NK cells could limit the immune-mediated tissue damage (Paya et al. 1989; Fairweather et al. 2001). On the other hand, a vigorous attack towards infected autologous cells could result in significant destruction of the target tissues (Flodstrom et al. 2002), which could lead to the release of self-antigens. As the inflammatory milieu caused by the infection may induce the priming of adaptative immune response, this can cause to the activation of normally quiescent autoreactive lymphocytes reacting to the recently spread self-epitopes.

Curiously, several small studies during the 80s have shown a reduced number and/or function of NK cells in the peripheral blood of patients with autoimmune diseases such as multiple sclerosis, rheumatoid arthritis, Sjogren syndrome and T1D (Hoffman 1980; Herold et al. 1984; Negishi et al. 1986; Lorini et al. 1994; French and Yokoyama 2004). In many of these studies, however, NK cell frequency was established based on non-specific markers that did not exclude NKT or other cells from the counts. NKT cells are T regulatory cells involved in the development of peripheral tolerance that share some receptors with NK cells but, differently from NK cells, present CD3 on their surface.

A reduced frequency or function of NK cells in the peripheral blood of patients with autoimmune diseases could represent a primary defect involved in the pathogenesis of the disease or a secondary effect of the disease itself or its treatment. In many cases, the treatment of autoimmune diseases includes corticosteroids. Although it has been shown recently that these drugs may reduce NK killing capacity (Mavoungou et al. 2005), studies in recently diagnosed patients with SLE and dermatomyositis in whom corticosteroid treatment had not been started show that the NK dysfunction cannot be attributed to the treatment (Yabuhara et al. 1996; O'Gorman et al. 2002). Although a deficiency of NK cells could be involved in the appearance of autoimmune diseases in general, interestingly, the main clinical characteristics of patients with complete and selective NK cells deficiency are not autoimmune diseases, but recurrent infections, especially the ones caused by herpesvirus (Biron et al. 1989). On the other hand, patients with NK related lymphocytosis frequently present autoimmune diseases (Tefferi et al. 1994).

Recently, it has been recognized that certain expression patterns of activating and/or inhibitory receptors on NK cells may be linked to the development of autoimmune diseases. A predominance of KIR activating receptors over inhibitory ones was reported in psoriatic arthritis (Martin et al. 2002). An increased expression of MICA in the sinovium of patients with reumathoid arthritis was also shown (Groh et al. 2003).

### NK cells in mouse models of type 1 diabetes

The two most intensively studied rodent models of autoimmune T1D are the nonobese diabetic mouse (NOD) and the BioBreeding (BB) rat. Both develop autoimmune mediated destruction of the pancreatic β cells after a variable period of insulitis similarly to human T1D. These animal models have been extremely important to help us elucidate the mechanisms involved in the development of T1D. The early stages of the disease process leading to T1D are characterized by insulitis, followed by β cell destruction in the later stages (Kelly et al. 2003). When most insulin-secreting cells are lost, T1D usually becomes clinically evident. The rate of progression from insulitis to T1D is variable and can range from a rapid destruction with a very early onset of the disease to a slow process with a late and quite often insidious clinical presentation. The precise mechanisms involved in the development of insulitis and its progression to necrosis and massive β cell destruction is still yet to be elucidated. Autoreactive T-cells have been shown to have a critical role in this process (Eisenbarth 1986). Although autoantibodies against the major antigens related to T1D are produced, they seem to be a consequence of β cell destruction and a wide spread of antigens present on these cells (Baekkeskov 1982).

A link between the development of diabetes and NK cells was suggested in the 80s. At that time, it was shown that, in diabetes prone BB rat, splenic NK cells were capable of destroying pancreatic islet cells in these animals (MacKay et al. 1986; Koevary 1988; Nakamura et al. 1990). More recently, the potential of NK cells to destroy islet cells was also demonstrated in NOD mice (Flodstrom et al. 2002). However, NK cells do not seem to be essential for the development of T1D, at least in animal models. Although the depletion of NK cells prevented the development of T1D in mice treated with streptozotocin (Maruyama et al. 1991a) and cyclophosphamide (Maruyama et al. 1991b), it did not result in similar prevention in mice that spontaneously develop T1D (Ellerman et al. 1993; Sobel et al. 1995) or in BB rats (Edouard et al. 1993; Ellerman et al. 1993). The role of NK cells in the pathogenesis of T1D could be a modulation of the intensity and aggressiveness of the β cell destructive process. Indeed, Poirot et al. (2004) have demonstrated a higher frequency of NK cells in the pancreatic infiltrate of BDC 2.5 transgenic mice with a B6.H-$2^{g7}$ genetic backgound, known to present a rapid progression from insulitis to T1D, than in BDC 2.5 transgenic mice with a NOD genetic background, which present innocuous insulitis with a rare and slow progression of the disease a higher frequency of NK cells in the pancreatic infiltrate of BDC2.5/B6.H-$2^{g7}$ transgenic mice. Flodstrom et al. (2002) suggested that NK cells could have a particularly important role in T1D induced by virus. According to this hypothesis, pancreatic β cells could have an abnormal response to IFN that would render them susceptible to viral infections and subsequent cell death induced by the NK cells. This could trigger T1D not only directly, causing β cell lysis, but also indirectly, as in genetic predisposed individuals the NK mediated damage could contribute to the release of self-antigens that could prime autoreactive T cells and trigger the disease.

Systemic abnormalities of NK cells have also been described in animal models for T1D. It has been shown that diabetes-prone BB/W rats have an increased frequency and activity of NK cells when compared with diabetes resistant rats of the same species (Woda and Biron 1986), although the opposite has been observed when intestinal NK cells were studied (Todd et al. 2004). Numeric and functional abnormalities in NK cells have also been reported in NOD mice and been implicated in the etiology of T1D (Kataoka et al. 1983; Poulton et al. 2001; Johansson et al. 2004). Poulton et al. (2001) have found a decreased numbers of peripheral NK cells in these animals with an increased frequency in the bone marrow, suggesting that a defect in NK export could be involved. Kataoka et al. (1983) described a depression of natural killer activity in 12-week-old NOD mice. A particularly interesting abnormality in NOD mice is a deficient activity of the receptor NKG2D in NK cells. In these animals, upon activation NK cells from NOD mice but not from C57BL/6 mice expressed NKG2D ligands, which resulted in downregulation of the receptor NKG2D. It is not yet known whether this abnormality is involved in the induction of diabetes in NOD mice (Ogasawara et al. 2003). Interestingly, a study performed by the same authors has shown that activation of NKG2D in CD8 + cells is essential for the progression from insulitis to diabetes in NOD mice (Ogasawara et al. 2004).

### NK cells in humans with type 1 diabetes

A few small studies in the 80s and 90s have shown a reduction of the frequency of NK cells in the peripheral blood in patients with T1D, especially in the ones with recent onset (Chandy et al. 1984; Herold et al. 1984; Pozzilli et al. 1984; Gupta et al. 1986; Wilson et al. 1986; Hussain et al. 1987). A few authors, however, have found a numeric deficiency of NK cells independently of the disease duration and suggested that this abnormality could be persistent and possibly genetically determined (Hussain et al. 1987). On the other hand other studies performed at that time did not find any abnormality in the frequency of NK cells in the peripheral blood (Herold et al. 1984; Scheinin et al. 1990). Two important concerns with those early studies are the small numbers of patients included and the use of non-specific markers such as H25, Leu7 (CD57) and Leu11a (CD16) (Scheinin et al. 1990; Baxter and Smyth 2002) to identify the NK cells. H25 is no longer available, but in any case bound to T cells in addition to NK cells. While Leu 7 (CD57) is not only also expressed in T cells but also absent on some NK cells, Leu11a (CD16) is present on monocytes, macrophages and some granulocytes in addition to NK cells. Recently, we have confirmed with a larger sample and more specific markers at the Joslin Diabetes Center (Boston, MA) that patients with recently diagnosed T1D have a slight reduction in the frequency of NK cell in the peripheral blood when compared to controls or patients with long-standing disease (Rodacki et al. 2006). However, the biological relevance of this finding is questionable, since the difference was quite modest. Nevertheless, this slight reduction could be related to the existence of insulinopenia or, alternatively, to a polarization of NK cells to the pancreas as a contributing factor for the β-cell destruction.

Functional abnormalities have also been reported in NK cells of patients with T1D. A reduced lytic capacity, as determined by cytotoxicity assays, was also reported in those earlier studies, both in recently diagnosed patients and/or the ones with long-standing T1D (Negishi et al. 1986; Lorini et al. 1994). As for the numeric abnormalities, these results were not universally confirmed (Nair et al. 1986; Hussain et al. 1987). In our recent analysis at the Joslin Diabetes Center, there was a reduced surface expression of the activating receptors NKp30 and NKp46 as well as lower mRNA levels of IFNg and perforin in NK cells of patients with long-standing T1D, but not in those recently diagnosed, when compared to controls without T1D. This indicates that there is a reduction in the activity of NK cells in patients with long-standing T1D but not in individuals with recent onset disease, probably as a consequence of the disease rather than a cause. The discrepancy between these results might be related to the criteria used to define the recent-onset group, varying from within 1 month to up to 1 year after the diagnosis. In our cohort, all recently diagnosed patients were tested within the first month of the disease.

The reduced activity of NK cells in patients with long-standing T1D but not in those recently diagnosed suggests that this could be a consequence of the disease. Either a direct effect in the NK cells or an abnormality that could interfere in their function could be potentially implicated, such as an impaired secretion of cytokines capable of interfering in NK cells function or alteration in ligands to the NK cells activating receptors. It is not yet known if the abnormalities in NK cell function have any influence in the risk of infectious diseases or neoplasias in patients with T1D.

Lanier et al. had demonstrated that activated NK cells in NOD mice displayed a low level of NKG2D. We compared the expression of this receptor in patients with T1D and controls and found a similarly reduced expression of NKG2D in the first group, independently of the duration of the disease (Rodacki et al. 2006). This suggests that there is a deficiency of NKG2D expression not only in NOD mice but also in humans with T1D. It is possible that this abnormality is involved in the induction of T1D.

A few studies have evaluated KIR genes in patients with T1D. While Van der Slik et al. (2003) have shown a predominance of genes encoding activating KIR genes in patients with T1D when compared to controls. Nikitina-Zake et al. (2004) have demonstrated that KIR2DL2, 2DS2 e 2DS3 were more prevalent in patients with T1D than controls without diabetes. These results suggest that certain KIR genes might influence the susceptibility to T1D, which would be reasonable since the KIR repertoire determines self-tolerance in NK cells and also influence the activity of T cells, both autoreactive and regulatory (Vilches and Parham 2002). It is possible that increased frequency of certain KIRs might be associated with autoreactivity in T1D. In our analysis, we confirmed the association between KIR2DS3 and T1D and also found interesting associations between this gene and HLA class II linked to T1D susceptibility. While HLA DR B1*03 was negatively associated with KIR2DS3, for HLA DR B1*04 a positive association was found (Rodacki et al. 2006). Combinations of HLA and KIR genes have been linked with susceptibility of autoimmune diseases (Rajagopalan and Long 2005).

### Conclusion

Abnormalities in the frequency and activity of NK cells have been described both in animal models and patients with T1D. Some of these alterations are linked to its onset while others seem to be a consequence of the disease. The elucidation of the exact role of NK cells in the pathogenesis of T1D is important in order to explore whether possible related immune interventions may affect the risk of T1D or delay its age of presentation. It is also important to clarify if NK abnormalities are involved in the risk of infections or neoplasia in patients with T1D and if any intervention can be used to correct this problem.

### Acknowledgements

We thank Diane Mathis and Christophe Benoist for mentoring Melanie Rodacki in the most recent analysis of NK cells in patients with T1D at the Joslin Diabetes Center (Boston, MA, USA).

### References

Andre P, Castriconi R, Espeli M, et al. 2004. Comparative analysis of human NK cell activation induced by NKG2D and natural cytotoxicity receptors. Eur J Immunol 34:961–971.

Arnon TI, Lev M, Katz G, et al. 2001. Recognition of viral haemagglutinins by NKp44 but not NKp30. Eur J Immunol 31:2680.

Backstrom E, Kristensson K, Ljunggren HG. 2004. Activation of natural killer cells: Underlying molecular mechanisms revealed. Scand J Immunol 60(1–2):14–22.

Baekkeskov S. 1982. Autoantibodies in newly diagnosed diabetic children immunoprecipitate human pancreatic islet cell proteins. Nature 167:169.

Baxter A, Smyth MJ. 2002. The role of NK cells in autoimmune disease. Autoimmunity 35(10):1–14.

Biron CA, Byron KS, Sullivan JL. 1989. Severe herpesvirus infections in adolescents without natural killer cells. N Engl J Med 320:1731–1735.

Bloushtain N, Qimron U, Bar-Ilan A, et al. 2004. Membrane-associated heparin sulfate proteoglycans are involved in the recognition of cellular targets by NKp30 and NKp46. J Immunol 173:2392–2401.

Chandy KG, Charles MA, Buckingham B, et al. 1984. Deficiency of monoclonal antibody (Leu7) defined NK cells in newly diagnosed insulin-dependent diabetes mellitus. Immunol Lett 8(2):89–91.

Colucci F, Caligiuri MA, DiSanto JP. 2003. What does it take to make a natural killer? Nat Rev Immunol 3:413–425.

Edouard P, Hiserodt J, Plamondon C, et al. 1993. CD8+ T cells are required for adoptive transfer of the BB rat diabetic syndrome. Diabetes 42:390–397.

Eisenbarth GS. 1986. Type 1 diabetes mellitus: A chronic autoimmune disease. N Engl J Med.

Ellerman K, Wrobleski M, Rabinovitch A, et al. 1993. Natural killer cell depletion and diabetes mellitus in the BB/Wor rat (revisited). Diabetologia 36(7):596–601.

Fairweather D, Kaya Z, Shellam GR, et al. 2001. From infection to autoimmunity. J Autoimmun 16:175–186.

Flodstrom M, Shi FD, Ljunggren H-G. 2002. The natural killer cell—friend or foe in autoimmune disease? Scan J Immunol 55(5):432–441.

Flodstrom M, Maday A, Balakrishna D, et al. 2002. Target cell defense prevents the development of diabetes after viral infection. Nat Immunol 3:373–382.

French AR, Yokoyama WM. 2004. Natural killer cells and autoimmunity. Arthritis Res Ther 6:8–14.

Groh V, Bruhl A, El-Gabalawy H, et al. 2003. Simulation of T cell autoreactivity by anomalous expression of NKG2D and its ligands in rheumatoid arthritis. Proc Natl Acad Sci USA 100:9452–9457.

Gupta S, Charles MA, Waldeck N, et al. 1986. Multiparameter immunologic studies in patients with newly diagnosed type 1 insulin-dependent diabetes mellitus. Diabetes Res 3(5): 225–229.

Hansson M, Kiessling R, Andresson B. 1981. Human fetal thymus and bone marrow contain target cells for natural killer cells. Eur J Immunol 11:8–12.

Herold KC, Huen A, Goud L, et al. 1984. Alterations in lymphocyte subpopulations in type 1 (insulin-dependent) diabetes mellitus: Exploration of possible mechanisms and relationships to autoimmune phenomena. Diabetologia 27(Suppl 1):102–105.

Hoffman T. 1980. Natural killer function in systemic lupus erythematosus. Arthritis Rheum 23:30–35.

Hussain MJ, Alviggi L, Millward BA, et al. 1987. Evidence that the reduced number of natural killer cells in type 1 diabetes may be genetically determined. Diabetologia 30:907–911.

Janeway CA, Travers P, Walport M, Shlomchik MJ. 2005. An introduction to immunobiology and innate imunity. In: Janeway CA, Travers P, Walport M, Shlomchik MJ, editors. Immunobiology. 6th ed. New York, NY: Ed Garland Science Publishing. p 1–35.

Johansson SE, Hall H, Bjorklund J. 2004. Broadly impaired NK cell function in non-obese diabetic mice is partially restored by NK cell activation *in vivo* by IL12/IL18 *in vitro*. Int Immunol 16(1):1–11.

Kataoka S, Satoh J, Fujiya H, et al. 1983. Immunologic aspects of nonobese diabetic mouse (NOD). Abnormalities of cellular immunity. Diabetes 32(3):247–253.

Kelly MA, Rayner ML, Mijovic CH. 2003. Molecular aspects of type 1 diabetes. Mol Pathol 1:10.

Koevary SB. 1988. *In vitro* natural killer cell activity in the spontaneously diabetic BB/W rat: Effects of serum on lysis of insulinoma cells. Diabetes Res 8(2):77–84.

Kos FJ, Engeman EG. 1996. Immune regulation: A critical link between NK cells and CTLs. Immunol Today 17:174–176.

Lanier LL, Bakker ABH. 2000. The ITAM-bearing transmembrane adaptor DAP12 in lymphoid and myeloid cell function. Immunol Today 21(12):611–614.

Lorini R, Moretta A, Valtorta A, et al. 1994. Cytotoxic activity in children with insulin-dependent diabetes mellitus. Diabetes Res Clin Pract 23:37–42.

MacKay P, Jacobson J, Rabinovitch A. 1986. Spontaneous diabetes mellitus in the biobreeding/Worcester rat. Evidence *in vitro* for natural killer lysis of islet cells. J Clin Invest 77:916–924.

Mandelboim ON, Lieberman M, Lev L, et al. 2001. Recognition of haemagglutinins on virus-infected cells by NKp46 activates lysis by human NK cells. Nature 409:1055.

Martin MP, Nelson G, Lee JH, et al. 2002. Cutting edge: Susceptibility to psoriatic arthritis: Influence of activating killer Ig-like receptor genes in the absence of specific HLA-C alleles. J Immunol 169:2818–2822.

Maruyama T, Watanabe K, Yanagawa T, et al. 1991. The suppressive effect of anti-asialo GM1 antibody on low-dose streptozotocin-induced diabetes in CD-1 mice. Diabetes Res 16(4):171–175.

Maruyama T, Watanabe K, Takei I, et al. 1991. Anti-asialo GM1 antibody suppression of cyclophosphamide-induced diabetes in NOD mice. Diabetes Res 17(1):37–41.

Mavoungou E, Bouyou-Akotet MK, Kremsner PG. 2005. Effects of prolactin and cortisol on natural killer (NK) cell surface expression and function of human natural cytotoxicity receptors (NKp46 NKp44 and NKp30). Clin Exp Immunol 139(2): 287–296.

Middleton D, Williams F, Halfpenny IA. 2005. KIR genes. Transpl Immunol 14(3–4):135–142.

Moretta L, Moretta A. 2004. Unravelling natural killer cell function: Triggering and inhibitory human NK receptors. EMBO J 23:255–259.

Moretta L, Biassoni R, Bottino C, et al. 2002. Natural killer cells: A mistery no more. Scand J Immunol 55:229–232.

Morse RH, Seguin R, McCrea EL, et al. 2001. NK cell-mediated lysis of autologous human oligodendrocytes. J Neuroimmunol 116:107–115.

Nair MP, Lewis EW, Schwartz SA. 1986. Immunoregulatory dysfunctions in type I diabetes: Natural and antibody-dependent cellular cytotoxic activities. J Clin Immunol 6(5):363–372.

Nakamura N, Woda BA, Tafuri A, et al. 1990. Intrinsic cytotoxicity of natural killer cells to pancreatic islets *in vitro*. Diabetes 39:836–843.

Negishi K, Waldeck N, Chandy G, et al. 1986. Natural killer cell and islet killer cell activities in type 1 (insulin-dependent) diabetes. Diabetologia 29(6):352–357.

Nikitina-Zake L, Rajalingham R, Rumba I, et al. 2004. Killer cell immunoglobulin-like receptor genes in latvian patients with type 1 diabetes mellitus and healthy controls. Ann NY Acad Sci 1037:161–169.

Ogasawara K, Hamerman JA, Hsin H. 2003. Impairment of NK cell function by NKG2D modulation in NOD mice. Immunity 18(1):41–51.

Ogasawara K, Hamerman JA, Ehrlich LR, et al. 2004. NKG2D blockade pervents autoimmune diabetes in NOD mice. Immunity 21(3):303–304.

O'Gorman M, Smith R, Garrison A, et al. 2002. Lymphocyte subsets in peripheral blood from newly diagnosed, untreated patients with juvenile dermatomyositis are associated with disease activity scores (DAS). Arthritis Rheum 46(9):S490.

Paya CV, Patick AK, Leibson PJ, et al. 1989. Role of natural killer cells as immune effectors in encephalitis and demyelination induced by Theiler's virus. J Immunol 143:95–102.

Pende D, Parolini S, Pessino A, et al. 1999. Identification and molecular characterization of NKp30, a novel triggering receptor involved in natural cytotoxicity mediated by human natural killer cells. J Exp Med 190:1505–1516.

Poirot L, Benoist C, Mathis D. 2004. Natural killer cells distinguish innocuous and destructive forms of pancreatic islet autoimmunity. Proc Natl Acad Sci 101(21):8102–8107.

Poulton LD, Smyth MJ, Hawke CG, et al. 2001. Cytometric and functional analyses of NK and NKT cell deficiencies in NOD mice. Int Immunol 13(7):887–896.

Pozzilli P, Sensi M, Al-Sakkaf L, et al. 1984. Prospective study of lymphocyte subsets in subjects genetically susceptible to type 1 (insulin-dependent) diabetes. Diabetologia 27(Suppl): 132–135.

Rajagopalan S, Long EO. 2005. Understanding how combinations of HLA and KIR genes influence disease. J Exp Med 201(7):1025–1029.

Raulet DH. 2004. Interplay of natural killer cells and their receptors with the apative immune response. Nat Immunol 5(10): 996–1002.

Raulet DH. 2003. Roles of the NKG2D immunoreceptor and its ligands. Nat Rev 3:781–790.

Rodacki M, Laffel L, Butty V. et al. 2006. In press. Frequency and activation state of natural killer cells in patients with type 1 diabetes. Diabetes 55(Suppl. 1): A1198.

Scheinin T, Maenpaa J, Kontiainen S. 1990. Immune responses to insulin and lymphocyte subclasses at diagnosis of insulin-dependent diabetes and one year later. Immunobiology 180(4–5):431–440.

Shi FD, Wang HB, Li H, et al. 2000. Natural killer cells determine the outcome of B cell-mediated autoimmunity. Nat Immunol 1:245–251.

Sinkovics JG, Horvath JC. 2005. Human natural killer cells: A comprehensive review. Int J Oncol 27(1):5–47.

Sivori S, Pende D, Bottino C, et al. 1999. NKp46 is the major triggering receptor involved in the natural cytotoxicity of fresh and cultured human NK cells. Correlation between surface density of NKp46 and natural cytotoxicity against autologous, allogeneic and xenogeinic target cells. Eur J Immunol 29:1656–1666.

Slik AR, Koeleman BPC, Verduijn W, et al. 2003. KIR in type 1 diabetes: Disparate distribution of activating and inhibitory natural killer cell receptors in patients versus HLA matched control subjects. Diabetes 52:2639–2642.

Slik AR, Koeleman BPC, Verduijn W, et al. 2003. KIR in type 1 diabetes: Disparate distribution of activating and inhibitory natural killer cell receptors in patients versus HLA matched control subjects. Diabetes 52:2639–2642.

Sobel DO, Azumi N, Creswell K, et al. 1995. The role of NK cell activity in the pathogenesis of poly I:C accelerated and spontaneous diabetes in the diabetes prone BB rat. J Autoimmun 8(6):843–857.

Tefferi A, Li CY, Witzig TE, et al. 1994. Chronic natural killer lymphocytosis: A descriptive clinical study. Blood 84: 2721–2725.

Todd DJ, Forsberg EM, Greiner DL. 2004. Deficiencies in gut NK cell number and function precede diabetes onset in BB rats. J Immunol 172(9):5356–5362.

Vilches C, Parham P. 2002. KIR: Diverse, rapidly evolving receptors of innate and adaptive immunity. Annu Rev Immunol 20: 217–251.

Vilches C, Parham P. 2002. KIR: Diverse, rapidly evolving receptors of innate and adaptive immunity. Annu Rev Immunol 20:217–251.

Wilson RG, Anderson J, Shenton BK, et al. 1986. Natural killer cels in insulin-dependent diabetes mellitus. Br Med J 293:244–245.

Woda BA, Biron CA. 1986. Natural killer cell number and function in the spontaneously diabetic BB/W rat. J Immunol 137(6): 1860–1866.

Yabuhara A, Yang FC, Nakazawa T, et al. 1996. A killing defect of natural killer cells are an underlying immunologic abnormality in childhood systemic lupus erythematous. J Rheumatol 23: 171–177.

